# Characteristics of footwear worn by people with systemic lupus erythematosus: a comparison with age- and sex-matched healthy controls: a pilot study

**DOI:** 10.1186/s13047-018-0280-3

**Published:** 2018-07-05

**Authors:** Sarah Stewart, Monique Keys, Angela Brenton-Rule, Ashok Aiyer, Nicola Dalbeth, Keith Rome

**Affiliations:** 1 Department of Podiatry, Health & Rehabilitation Research Institute, Auckland University of Technology, Private Bag 92006, Auckland, 1142 New Zealand; 2 Faculty of Medical and Health Sciences, The University of Auckland, Private Bag 92019, Auckland, 1142 New Zealand; 3 Department of Rheumatology, Auckland District Health Board, P.O. Box 92189, Auckland, New Zealand

**Keywords:** Systematic lupus erythematosus, Footwear, Foot pain

## Abstract

**Background:**

To determine characteristics of footwear worn by people with systematic lupus erythematosus (SLE).

**Methods:**

Twenty-two people with SLE and twenty matched healthy controls participated in a cross-sectional study. Objective assessments of footwear included: fit, style, structure, motion control, cushioning, and wear. Footwear was classified as poor, average or good based on a standardised tool. Participants completed 100mm visual analogue scales for foot pain and footwear comfort and suitability. Participants with SLE were asked to indicate which footwear features were important to them using a validated checklist.

**Results:**

No differences were observed between groups for footwear fit, age, style, heel height, forefoot flexion or cushioning (all *P*>0.05). Compared to controls, a greater number of participants with SLE wore shoes with worn tread (65% vs. 91%, *P*=0.041), wore shoes with a lower motion control scale (median: 5.0 vs. 1.0, *P*=0.003), and rated their footwear as less comfortable (median: 90mm vs. 78mm, *P*=0.024) and less suitable (median: 88mm vs. 76mm, *P*=0.030). Participants with SLE experienced greater foot pain than controls (median: 17mm vs. 0mm, *P*=0.038). Comfort (95%), fit (95%) and style (86%) were identified as the most important footwear features by people with SLE.

**Conclusions:**

Compared to control participants, people with SLE wear shoes that are more worn and lack motion control. They also report greater foot pain and report their shoes to be less comfortable and suitable. These findings highlight the need for a further focus on the role of footwear in the management of foot problems in people with SLE.

## Background

SLE is a multisystem autoimmune disease that manifests in many systems, including vascular, neurological, musculoskeletal and cutaneous tissues [1–3]. SLE frequently affects peripheral structures, with the foot and ankle region being common areas of involvement [4]. Foot problems in people with SLE include impaired peripheral blood flow [5, 6], peripheral neuropathy [7], toe deformities [8, 9], joint swelling [10, 11], skin and nail changes [8, 10, 12] and gait changes [10, 11, 13].

Footwear is important in providing protection, accommodating deformity and assisting function [14]. Different footwear characteristics, including heel height, lack of support and poor fit, may result in increased pain as well as the development of musculoskeletal complications such as osteoarthritis and hallux valgus in otherwise healthy people , as well as foot pain and impairment in people with inflammatory arthritis, including gout [15] and rheumatoid arthritis[16]. Footwear difficulties have also been linked to psychosocial factors, including the aesthetic appearance of footwear which had been identified as a major limiting factors in footwear selection in women with rheumatic disease [17]. In fact, female patients with rheumatoid arthritis choose not to wear specialist therapeutic footwear due to the associated restrictions in clothing and contribution to social isolation [18]. In a recent survey, 52% of people with SLE reported difficulty wearing different shoes [11]. A subjective global podiatric assessment of footwear found that 48% of people with SLE wore shoes deemed as ‘inappropriate’, compared with 35% of control participants [8].

Despite the frequency of foot complications in people with SLE, and the association between footwear characteristics and foot problems in other inflammatory conditions, a detailed objective assessment of footwear has not yet been undertaken in people with SLE. The aim of this study was to describe, in detail, the characteristics of footwear worn by people with SLE.

## Methods

### Participants

This cross-sectional observational study involved a total of 42 participants, including 22 people with SLE and 20 age- and sex-matched healthy controls. Participants were recruited via convenience sampling and this sample size was determined by a fixed 10 week recruitment and data collection period (December 2017 to February 2018). Auckland University of Technology (AUT) Ethics Committee approved the study (AUTEC 16/209). All participants with SLE were diagnosed by a rheumatologist, and fulfilled the SLICC criteria for the classification of SLE [19]. Participants with SLE were recruited from secondary care rheumatology clinics in the Auckland region, New Zealand. Control participants were recruited from AUT staff through poster and newsletter advertising. Participants in both groups were included if they were > 20 years of age, were able to read English, and had no recent foot surgery or trauma, neuromuscular conditions or other rheumatic diseases. All participants provided written informed consent prior to the collection of data.

### Data collection

All participants attended a single clinical visit between the months of December 2017 to February 2018 (New Zealand summer). Participants were asked to wear the footwear they had worn most often over the previous month to the study visit. Demographic information and clinical history were obtained from participants during the study visit.

A footwear assessment tool was used to objectively examine the detailed footwear characteristics of the shoes worn by the participants during the study visit [14]. The footwear assessment tool is comprised of six components that cover fit, general features, general structure, motion control properties, cushioning and wear patterns. All components in this tool demonstrate high intra- and inter-rater reliability [14]. Each item from the motion control category was scored from a range of 0 to 11, in which footwear with a score of 11 was considered to possess optimal motion control properties.

In addition, footwear was classified as being poor, average or good using a footwear classification tool [20]. Poor footwear were classified as those which lacked support and sound structure, including sandals, flip-flops (jandals), slippers, mules and moccasins. Average footwear was comprised of hard or rubber-soled shoes and work boots and good footwear was classified as athletic shoes, walking shoes, therapeutic footwear or Oxford type shoes [20].

Participants were also asked to identify feature(s) of footwear that they considered important when they choose and wear shoes, using a validated checklist [21]. This checklist has been used to assess footwear features in other inflammatory arthropathies [22, 23] and includes items such as comfort, style, fit, support, cost, weight and colour. Self-perceived comfort and suitability of current footwear were assessed using 100 mm visual analogue scales (VAS). In addition, foot pain for the foot with the greatest pain at the time of the study visit was assessed using 100 mm VAS.

### Statistical analysis

All demographic and clinical data were described as mean (SD) for continuous data and n (%) for categorical data. To determine whether there were differences in footwear characteristics between groups, Wilcoxon signed rank tests (for non-parametric continuous data or ordinal data) and McNemar’s chi-square tests (for binary data) were used. All analyses was undertaken in SPSS v. 24.

## Results

Participant demographic and clinical data are summarised in Table 1. Participants were predominantly middle-aged females. Participants with SLE had a mean (SD) disease duration of 14 (10) years. Participants with SLE and controls were generally well matched for age, sex, ethnicity and body mass index.

**Table 1:** Participant demographic and clinical characteristics

	SLE	Control
N	22	20
Sex, female, n (%)	20 (91%)	18 (90%)
Age, years	51 (15)	51 (12)
Ethnicity, n (%)		
European	13 (59%)	17 (85%)
Māori	2 (9%)	1 (5%)
Pacific	1 (5%)	0 (0%)
Asian	6 (27%)	1 (5%)
Other	0 (0%)	1 (5)
Body mass index, kg/m^2^	27.1 (7.0)	26.3 (5.3)
SLE disease duration, years	14.3 (10.3)	-
SLEDAI-2K	12.0 (8.2)	-
Medications, n (%)		
Hydroxychloroquine	12 (55%)	
Immunosuppressive	10 (42%)	
NSAID	10 (45%)	
Prednisone	11 (50%)	
Comorbidities and complications of disease, n (%)		
Raynaud’s syndrome	9 (41%)	
Lupus nephritis	3 (14%)	
Hypertension	3 (14%)	
Cardiovascular disease	2 (9%)	
Dyslipidaemia	2 (9%)	
Sjögren syndrome	2 (9%)	
Osteoporosis	2 (9%)	
Fibromyalgia	1 (5%)	
Depression	2 (9%)	

Values are presented as mean (SD) unless otherwise indicated. SLEDAI-2K: systematic lupus erythematosus disease activity index – 2000; NSAID: non-steroidal anti-inflammatory drug

Table 2 summarises the footwear characteristics. There was no significant difference between footwear worn by people with SLE and controls in terms of fit, style, classification, longitudinal profile, forefoot flexion point and cushioning. A greater number of people with SLE wore shoes that had partly or fully worn tread, compared to controls (91% vs. 65%, *P* = 0.041). The most common footwear style worn by both groups was walking shoes (*n*=7 (32%) SLE, *n*=7 (35%) controls), followed by open-toed shoes, including sandals (*n*=6, (27%) SLE, *n*=5 (25%) controls) and flip flops (*n*=3 (14%) SLE, *n*=1 (5%) controls). The majority of participants with SLE wore shoes classed as poor (*n* = 14, 64%) however, this was not significantly different from controls (*n*=9 (45%), *P* = 0.47). The majority of shoes worn in both groups had no heel height and around one-third of participants wore shoes with forefoot flexion points distal or proximal to the metatarsal heads. Participants with SLE wore shoes with a lower motion control properties scale compared to controls (median: 1.0 vs. 5.0, *P* = 0.003).

**Table 2:** Footwear characteristics^a^

	SLE	Control	*P*
Appropriate fit in length	14 (66%)	14 (70%)	0.33
Appropriate fit in width	17 (77%)	18 (90%)	0.27
Age of shoe > 12 months	12 (55%)	8 (40%)	0.35
Partly or fully worn tread	20 (91%)	13 (65%)	**0.041**
Footwear style			0.34
Walking shoe	7 (32%)	7 (35%)	
Athletic shoe	0 (0%)	3 (15%)	
Boot	1 (5%)	1 (5%)	
Sandal	6 (27%)	5 (25%)	
High-heel	1 (5%)	0 (0%)	
Court	1 (5%)	0 (0%)	
Moccasin	0 (0%)	2 (10%)	
Flip flops	3 (14%)	1 (5%)	
Other	3 (14%)	1 (5%)	
Classification of footwear			0.47
Good	7 (32%)	10 (50%)	
Average	1 (5%)	1 (5%)	
Poor	14 (64%)	9 (45%)	
Longitudinal profile			0.37
Flat	13 (59%)	12 (60%)	
Small heel rise	7 (32%)	8 (40%)	
Large heel rise	2 (9%)	0 (0%)	
Forefoot flexion point			0.58
Level with MTPJs	15 (68%)	12 (60%)	
Proximal/distal to MTPJs	7 (32%)	8 (40%)	
Motion control properties scale (0-9), median (IQR)	1.0 (4.0)	5.0 (4.0)	**0.003**
Presence of cushioning			0.75
None	8 (36%)	6 (30%)	
Heel	2 (9%)	1 (5%)	
Heel and forefoot	12 (55%)	13 (65%)	
Footwear comfort VAS, mm, median (IQR)	78.0 (34.0)	90 (29.0)	**0.024**
Self-perceived footwear suitability VAS, mm, median (IQR)	76.0 (28.0)	88.0 (33.0)	**0.030**
Foot pain VAS, mm, median (IQR)	17.0 (62.5)	0.0 (10.5)	**0.038**

^a^Values are presented as n (%) unless otherwise specified. Bolded-p values indicate significant between group differences at *P* < 0.05. MTPJs: metatarsophalangeal joints; VAS: visual analogue scale

Participants with SLE rated their footwear as less comfortable (median: 78mm vs. 90mm, *P* = 0.024) and suitable (median: 76mm vs. 88mm, *P* = 0.030) compared to controls. Participants with SLE had greater foot pain compared to controls (median: 0mm vs. 17mm, *P* = 0.038). Table 3 shows the differences in features of footwear perceived as important or very important between people with SLE and controls. The most commonly identified factors for both groups were comfort (95% SLE vs 100% controls), fit (95% SLE vs 100% controls) and style (86% SLE vs 90% controls). Only ease to put on and take off the shoe was significantly different between groups (82% SLE vs 50% controls, *P* = 0.029).

**Table 3.** Difference in footwear features considered important to people with SLE and controls

	SLE	Control	*P*
Comfort	21 (95%)	20 (100%)	0.34
Fit	21 (95%)	20 (100%)	0.34
Style	19 (86%)	18 (90%)	0.72
Ease to put on/off	18 (82%)	10 (50%)	**0.029**
Heel height	17 (77%)	15 (75%)	0.86
Support	16 (73%)	15 (75%)	0.87
Non-slip	16 (73%)	11 (55%)	0.23
Colour	15 (68%)	13 (65%)	0.83
Material	14 (64%)	15 (75%)	0.43
Cost	12 (55%)	10 (50%)	0.77
Weight	10 (45%)	10 (50%)	0.77
Fastening	9 (41%)	7 (35%)	0.69

Data are presented as n (%). Bolded-p values indicate significant between group differences at *P* < 0.05.

## Discussion

This is the first study to undertake a comprehensive assessment of footwear worn by people with SLE. The findings show that overall, footwear characteristics are similar between people with SLE and age- and sex-matched controls in terms of fit, age, style, heel height, forefoot flexion and cushioning. However, people with SLE wore shoes that were more worn, lacked motion control properties and were reported to be less suitable and less comfortable compared to those worn by healthy volunteer controls.

People with SLE wore shoes with less motion control properties compared to controls. These properties include stiffness of the heel counter, which is important in controlling rearfoot motion, and sagittal and frontal plane stability which are importing in controlling motion through the midfoot [14]. Although the sample size was not powered to determine the relationship between foot characteristics and foot problems, prior studies have shown that adequate motion control is important in managing foot problems in people with rheumatoid arthritis and gout [15, 16] and is associated with lower foot-related pain and disability in people with gout [23].

Although the majority of participants with SLE wore shoes classed as poor, including sandals and flip flops, their shoe choice did not differ from the control participants. The frequent use of poor footwear by people with SLE observed in the current study is similar to a previous study of foot problems in people with SLE, which described a subjective assessment of footwear appropriateness [8]. It should be noted that the current study was conducted over summer, and previous research has shown seasonal variation in footwear worn by people with inflammatory arthritis, who frequently wear sandals in summer to prevent their feet over-heating [24].

Consistent with prior reports in rheumatoid arthritis [22] and gout [23], comfort and fit were highlighted by patients with SLE as being the most important footwear factors. Interestingly, people with SLE also rated their footwear as being less suitable and less comfortable compared to control participants. This may reflect the difficulty that people with SLE have in wearing different shoes [11]. A recent survey found that 27% of people with SLE reported that they would like to receive footwear advice, while 23% reported having received advice related to footwear [10]. Finding appropriate footwear has been identified as a major barrier to footwear-based treatment in people with inflammatory arthritis [18, 25, 26].

The results from this study should be considered in light of a number of limitations. Firstly, the study involved a small number of participants which reduced the power of the study. Secondly, this study was conducted over summer in New Zealand and the results may vary in other countries and climates due to seasonal variation in footwear. The small sample size meant the study was underpowered to assess associations between footwear and foot problems in participants with SLE. Future studies may determine the relationship between characteristics of footwear worn by people with SLE and their foot pain, foot function and foot deformity. Future work may also assess the efficacy of footwear education on footwear habits, management of foot problems and reduction of foot pain in people with SLE.

## Conclusions

In summary, this study has shown that compared to age- and sex-matched healthy volunteer controls, people with SLE wear shoes that are more worn and lack motion control properties. People with SLE also reported greater foot pain, and perceived their footwear to be less comfortable and less suitable. These findings may highlight the need for further consideration of the role of appropriate footwear and footwear advice in managing foot problems in people with SLE.

## Abbreviations

SLE: Systemic lupus erythematosus
VAS: Visual analogue scale
